# Influence of Alkali Treatment of Jatropha Curcas L. Filler on the Service Life of Hybrid Adhesive Bonds under Low Cycle Loading

**DOI:** 10.3390/polym15020395

**Published:** 2023-01-12

**Authors:** Viktor Kolář, Petr Hrabě, Miroslav Müller, Monika Hromasová, David Herák, Hadi Sutanto

**Affiliations:** 1 Department of Material Science and Manufacturing Technology, Faculty of Engineering, Czech University of Life Sciences Prague, 165 00 Prague, Czech Republic; 2 Department of Electrical Engineering and Automation, Faculty of Engineering, Czech University of Life Sciences Prague, 165 00 Prague, Czech Republic; 3 Department of Mechanical Engineering, Faculty of Engineering, Czech University of Life Sciences Prague, 165 00 Prague, Czech Republic; 4 Department of Mechanical Engineering, Faculty of Engineering, Atma Jaya Catholic University of Indonesia, Jakarta 129 30, Indonesia

**Keywords:** NaOH treatment, oilseed cake, natural filler, material utilization, single-lap bond

## Abstract

The aim of this research was to evaluate the effect of untreated and 5% aqueous NaOH solution-treated filler of the plant Jatropha Curcas L. on the mechanical properties of adhesive bonds, especially in terms of their service life at different amplitudes of cyclic loading. As a result of the presence of phorbol ester, which is toxic, Jatropha oilseed cake cannot be used as livestock feed. The secondary aim was to find other possibilities for the utilization of natural waste materials. Another use is as a filler in polymer composites, that is, in composite adhesive layers. The cyclic loading of the adhesive bonds was carried out for 1000 cycles in two amplitudes, that is, 5–30% of the maximum force and 5–50% of the maximum force, which was obtained by the static tensile testing of the adhesive bonds with unmodified filler. The static tensile test showed an increase in the shear strength of the adhesive bonds with alkali-treated filler compared to the untreated filler by 3–41%. The cyclic test results did not show a statistically significant effect of the alkaline treatment of the filler surface on the service life of the adhesive bonds. Positive changes in the strain value between adhesive bonds with treated and untreated filler were demonstrated at cyclic stress amplitudes of 5–50%. SEM analysis showed the presence of interlayer defects in the layers of the tested materials, which are related to the oil-based filler used.

## 1. Introduction

Adhesive bonding is widely used in many industries such as automotive and aerospace [1]. Nowadays and with the development of adhesive science, the use of this type of bonding has become more attractive due to its higher peel and shear strengths, as well as the allowable tensile strength up to the failure of the bonded layer [2,3]. Adhesive bonds have some advantages over traditional bonds (welded, bolted, or riveted), such as lower weight, more uniform stress distribution, absence of damage to the bonded parts, ease of fabrication, ability to bond dissimilar materials, etc. [4,5]. However, adhesive bonding provides not only a bonding function but also a support function, that is, sealing, clamping, and securing [6,7].

Jatropha Curcas L. is a small tree or shrub that grows rapidly and is widespread in tropical Mexico, Central America, Africa, and Asia [8,9]. The seeds of this tree are used to obtain oil by pressing. The pressing of oil from the seeds of Jatropha Curcas L. was studied by Kabutey et al. [10]. The use of oil has applications in medicine in the treatment of skin, respiratory, and digestive problems [11] or in the production of ‘jet biofuel’ [12]. Deeba et al. [13] have used Jatropha oil and deoiled seed husk to produce biodiesel and biogas. The waste produced after pressing oilseed plants is called oilseed cake. Oilseed cake is commonly used as feed for livestock [14] or as a source of protein for humans [15]. However, Jatropha Curcas L. is a toxic plant due to its phorbol ester content [11,16] and therefore oilseed cakes cannot be used for food or feed purposes. However, oilseed cakes can be used for energy applications such as pellets [17], for the production of biogas [18,19], or as filler in polymer composite materials. Shivamurthy et al. [18] used oilseed cake particulate filler in a polymer composite to evaluate the mechanical properties. Valášek [19] and Hrabě et al. [20] used the oilseed cake particle filler from Jatropha Curcas L. to investigate the abrasive wear of polymer composite.

The generation of waste, or the waste of material, is a global issue. The accumulation of waste leads to an increase in environmental pollution [21]. Agricultural wastes in the form of fibers, shells, chips, husks, powders, particles, etc., can be innovatively recycled and reused in various industries, that is, as a filler in polymer composite materials [22,23,24,25,26]. The advantages of using natural waste materials in composites include, for example, the low cost of waste materials and the reduction in the amount of materials that are landfilled or incinerated, thus producing fewer CO_2_ emissions [21]. The reuse of all materials generated during the processing of agricultural crops can increase the economic efficiency of the whole process and reduce the negative impact on the environment [27]. Therefore, the use of waste materials should be promoted and their role in different applications should be investigated in detail. However, the use of natural wastes carries risks such as differences in the size and shape, moisture content, and surface properties of the natural fillers obtained subsequently [21,28].

The surface properties of natural fillers have a significant effect on the mechanical properties of polymer composites because of their wetting by the matrix. The wettability of natural fillers by the matrix is a key factor that affects their properties. The reduced wettability (adhesion) of natural fillers, which usually reduces the shear strength of adhesive bonds, is one of the significant disadvantages of using natural fillers in polymer composites [29,30,31,32]. The reduced surface wettability of natural fillers can be minimized, e.g., by the chemical treatment of their surface in an aqueous NaOH solution, plasma surface treatment, etc. [33]. The alkaline action of the aqueous NaOH solution improves the surface microstructure of the natural material [34]. The improvement in surface texture is due to the removal of unwanted layers from the surface of the filler [35,36]. This involves the removal of cellulose, hemicellulose, and oils, which are the main causes of poor wettability in natural fillers. Surface modification leads to improved interactions at the interfacial interface, that is, the filler–matrix interface [37]. The improvement of the interactions leads to an improvement in the mechanical properties, especially the shear strength of the adhesive bonds [38].

The increasing use of polymer-based materials in various advanced engineering components and structures requires better fatigue properties of polymers [39]. Adhesive bonds are not yet completely reliable in critical structural bonds due to problems, such as the cracking of the polymer matrix, cyclic fatigue, uncertainty of long-term behavior, or greater variance of failure load values [40]. The fatigue behavior of polymers is affected by several parameters, such as cyclic loading amplitude, intensity, and frequency [41]. Consequently, microcracks, called polymer matrix cracking, can form in polymer composites. Cracking is characterized as the gradual accumulation of plastic deformation in materials that are subjected to cyclic loading with non-zero mean stress. The accumulation of plastic deformation is an important aspect of fatigue damage in materials [42,43]. Thus, microcracks can ultimately lead to overall material degradation and affect the long-term service life of polymer composite materials, that is, even adhesive bonds [41]. Sarac et al. [44] investigated single-lap bonds with a composite layer of nanoparticle-based adhesive, and the results show a positive effect on fatigue strength. Chu et al. [45] investigated organoclay (organo-modified montmorillonite) nanofiller and found a positive effect from extending the fatigue life of single-lap bonds, while the shear strength of these adhesive bonds increased by 60%.

The novelty of the work is the utilization of a secondary product from the processing of oilseeds (oilseed cake), which has limited the further utilization of the material due to its toxicity. The secondary product was obtained from a pressing process in Indonesia. Previous investigations have shown the positive effect of natural filler on the cyclic loading service life of hybrid adhesive bonds [36,46,47], which enables the extension of adhesive bonds subjected to low cyclic loading, that is, low cycle fatigue. Low cycle fatigue has a negative effect on adhesive bonds with pure adhesive. However, these studies did not address the issue of the alkaline surface treatment of the natural filler. Alkaline surface treatment and its effect is mainly investigated in research focused on the surface texture of fillers or increasing the static strength of polymer composites. This research focuses not only on the modification of the filler and its effect on the service life of hybrid adhesive bonds but also specifically on other applications of this secondary product.

The aim of this research was to evaluate the effect of untreated and 5% aqueous NaOH solution-treated filler of the plant Jatropha Curcas L. on the mechanical properties of adhesive bonds, especially in terms of their service life at different amplitudes of cyclic loading. The secondary aim was to find further possibilities for the material utilization of natural waste.

## 2. Materials and Methods

Oilseed cakes of Jatropha Curcas L. crop were used as filler. Oilseed cakes of whole kernels including husk, kernels without husk, and husk were used. The Jatropha oil seed cake contains 56.31% cellulose, 17.47% hemicellulose, 23.91% lignin and 1.5% ash. The chemical composition of Jatropha Curcas L. was further discussed in detail by Müller et al. [48]. Oilseed cakes were dried in a laboratory oven at 105 °C for 24 h, ground with a Retsch MM 400 oscillating mill (Retsch Verder s.r.o., Prague, Czech Republic), and then sorted into size fractions by sieve analysis. The resulting fraction size was determined using a 100 μm-mesh screen. The size of the particulate filler was measured with the Gwyddion program (version 2.49, David Nečas and Petr Klapetek, Brno University of Technology, Brno, Czech Republic) and was 55 ± 21 μm for whole kernels including the skin, 63 ± 14 μm for kernels without skin, and 78 ± 20 μm for skin.

**Figure 1:**
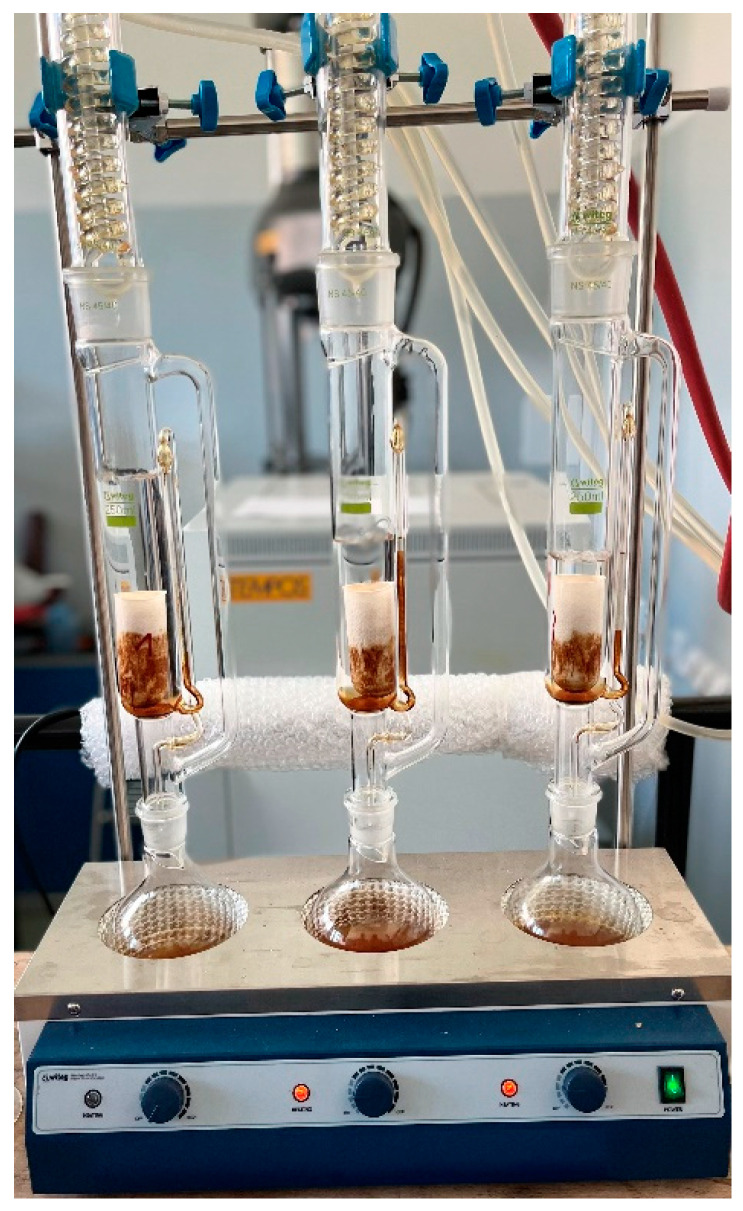
Soxhlet apparatus.

The residual oil content in oilseed cakes was determined using an automatic Soxhlet apparatus (Witeg Labortechnik GmbH, Wertheim, Germany) according to the manufacturer’s instructions [49]. The Soxhlet apparatus is shown in Figure 1.

Test samples of whole kernels, including the skin, kernels without skin, and skins, were ground to a powder and dried. The oil was extracted from the dried powder (10 g) with petroleum ether (Penta, s.r.o., Prague, Czech Republic) for 6 h at 60 °C. The oil was dried at 105 °C for 5 h to remove residual water and petroleum ether after extraction was completed [50]. The oil content was 26.78 ± 1.03% in whole kernel samples including the husk, 0.82 ± 0.07% in the skins, and 48.97 ± 0.17% in the kernels without the skin.

The alkaline treatment of the filler surface was carried out with 5% aqueous NaOH solution (Barvy a laky Hostivař a.s., Prague, Czech Republic) for 0.5 h. Table 1 shows the marking of the different types of fillers. Adhesive bonds also use this marking.

**Figure 2:**
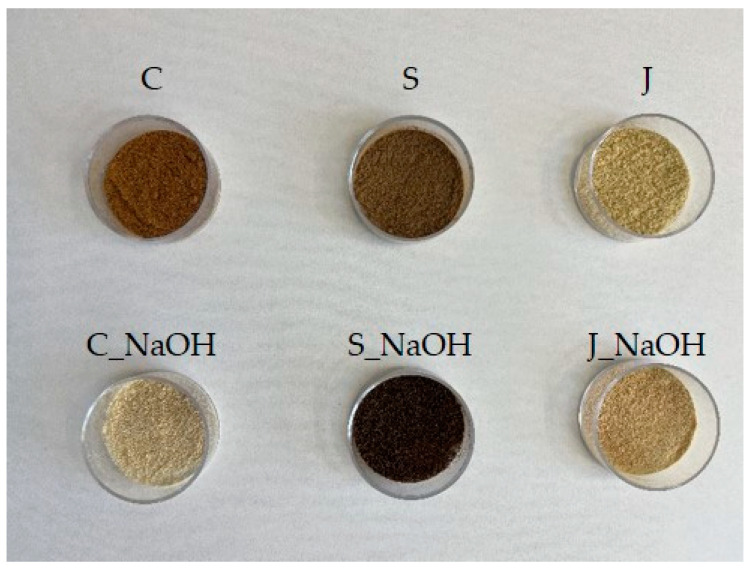
Oilseed cakes from Jatropha Curcas L. (filler) before and after alkaline surface treatment.

Figure 2 shows the diverse types of fillers before and after the alkaline treatment of their surfaces.

The matrix was a two-component epoxy resin Epoxy 1200 (CHS-Epoxy 324) (Havel Composites CZ s. r. o, Svésedlice, Czech Republic) with hardener P11 (Havel Composites CZ s. r. o, Svésedlice, Czech Republic) in a weight ratio of 100:7. The filler was added to the matrix at a concentration of 20 wt.%. This concentration of natural filler was shown to be optimal in terms of mechanical properties (service life) of the adhesive bonds exposed to cyclic loading [46,47].

**Figure 3:**
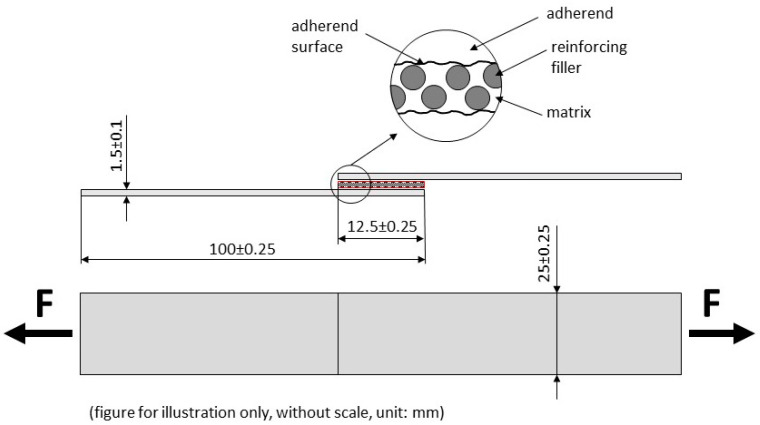
Scheme of adhesive bond according to EN 1465 [52].

Structural carbon steel S235J0 (Ferona a.s., Prague, Czech Republic) with a thickness of 1.5 ± 0.1 mm, length 100 ± 0.25 mm, and width 25 ± 0.25 mm was used as the adherend. The dimensions of the adherend were determined according to ČSN EN 1465 [51]. Figure 3 presents a schematic of the adhesive bonds, including their dimensions according to this standard.

The adherends were mechanically and chemically treated shortly before the application of the composite layer. The mechanical treatment of the adherend surfaces was carried out in a blasting booth with Garnet MESH 80 abrasive and the chemical treatment of the surfaces in an acetone bath. The surface of the treated adherends was subjected to roughness measurements using a Mitutoyo Surftest 301 profilometer (Mitutoyo Europe GmbH, Neuss, Germany), with Ra = 3.21 ± 0.24 μm and Rz = 11.28 ± 0.11 μm. The basic mechanical properties of the adherend are shown in Table 2.

**Figure 4:**
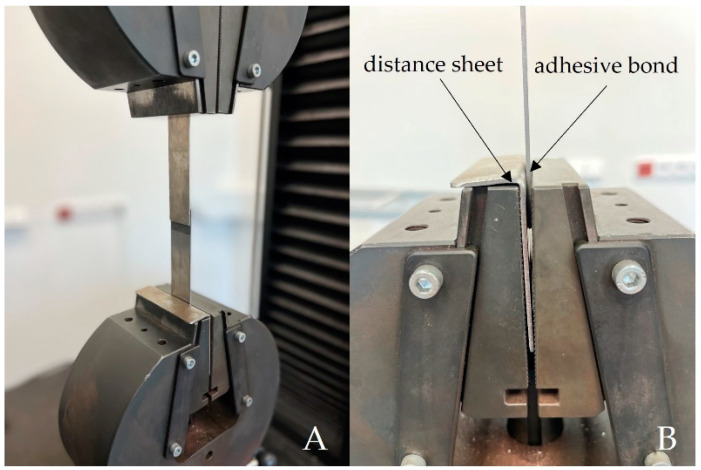
Jaws of LABTest 5.50 ST: (**A**): detailed view of the sample; (**B**): distance sheet to compensate for the bending moment of the adherend.

Mechanical properties testing was performed on the LABTest 5.50 ST universal testing machine (LABORTECH s.r.o., Opava, Czech Republic) with the measuring unit AST KAF 50 kN (LABORTECH s.r.o., Opava, Czech Republic) and the evaluation software Test&Motion (version 4.5.0.15, LABORTECH s.r.o., Opava, Czech Republic). Figure 4 shows the jaws of the LABTest 5.50 ST. Figure 4A presents a close-up view of the sample, and Figure 4B presents the distance sheet, which provides the compensation of the bending moment of the adherend acting on the adhesive bond when clamped in the jaws of the testing machine. The bending moment can negatively affect the strength of the adhesive bonds [54].

The methodology for the mechanical properties testing under cyclic loading, that is, shear strength and elongation at break, consisted of the determination of a reference value obtained during a static tensile test (ČSN EN 1465) from seven adhesive bonds marked C, S, and J at a test speed of 0.6 mm × min^−1^. The reference value corresponds to the maximum static force (Fmax) required to completely break the adhesive bond. Cyclic testing consisted of setting different amplitudes obtained as a percentage of the reference values (% of Fmax). The amplitudes of the C, S, and J adhesive bonds were applied to the C_NaOH, S_NaOH, and J_NaOH adhesive bonds to determine the effect of the alkaline treatment of the filler surface on their service life. A static test was also performed on the AB0 adhesive bond to compare the results of the tensile test with the adhesive bonds with the composite adhesive layer. The cyclic testing of the AB0 adhesive bonds was also carried out but only to verify the cyclic loading service life. The cyclic stress results of the AB0 adhesive bonds are not comparable to those of the composite adhesive bonds due to the different ranges of values of the individual amplitudes. Table 3 shows the basic settings of the cyclic tests, that is, the Fmax and the individual amplitudes of the cyclic loading.

Adhesive bonds were loaded for 1000 cycles at a speed of 6 mm × min^−1^. After the completion of 1000 cycles, a static tensile test was automatically followed until the complete failure of the adhesive bond at a speed of 0.6 mm × min^−1^. The static test was only performed when the 1000th cycle was completed. Otherwise, the test was terminated. The time delay at the lower and upper amplitude limits was set to 0.5 s. Each test series contained seven test specimens.

**Figure 5:**
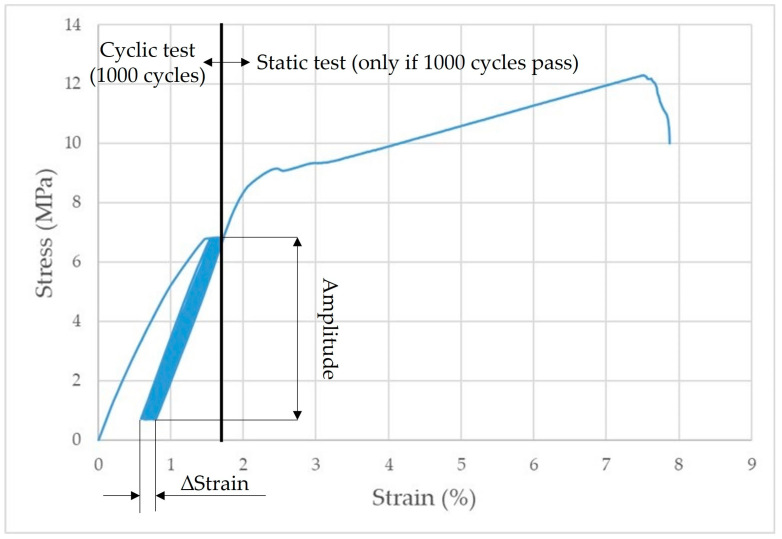
Principle of low cycle loading of adhesive bonds.

Figure 5 shows the principle of the low cycle loading of adhesive bonds.

Statistical evaluation of the experiments was performed by analysis of variance, that is, ANOVA F-test in STATISTICA (version 14.0.0.15, StatSoft CR, Prague, Czech Republic). Statistical testing evaluates the relationship between untreated and treated filler surface on the mechanical properties of the different variants of adhesive bonds. The statistical dependence at the 0.05 level of significance between the diverse types of adhesive bonds, that is, between C and C_NaOH, J and J_NaOH, S and S_NaOH, was evaluated. Hypothesis H_0_ was established and presents a statistically insignificant difference between the different variants of adhesive bonds (*p* > 0.05). Hypothesis H1 rejects hypothesis H0 and presents a statistically significant difference between the different variants of adhesive bonds (*p* < 0.05).

The SEM Analysis was performed using a MIRA 3 TESCAN electron microscope (Tescan Brno s.r.o., Brno, Czech Republic), that is, the used fillers, the interaction at the interfacial interface of filler/matrix, and the bonded material/adhesive composite layer were evaluated. The microscopic samples were coated with gold using a Quorum Q150R ES (Tescan Brno s.r.o., Brno, Czech Republic). The parameters of the SEM images can be seen from the bottom caption of the images at HV 5 kV using an Oxford SE detector.

## 3. Results and Discussion

**Figure 6:**
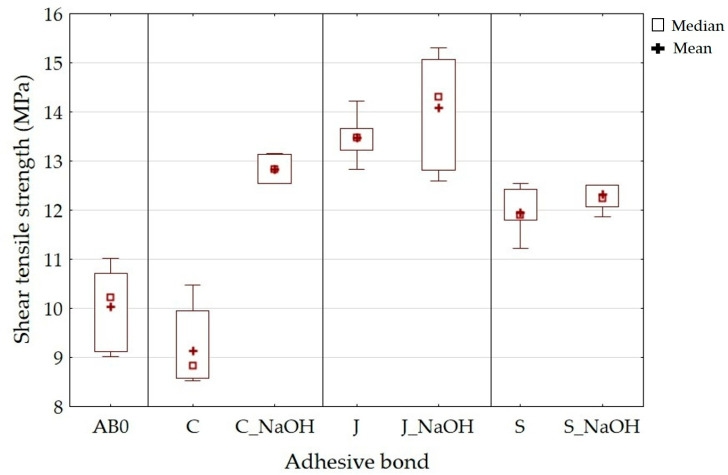
Static tensile test—shear tensile strength.

The results of the static shear tensile strength are shown in Figure 6. The results show the positive effect of the chemical surface treatment of the fillers on the shear tensile strength. There was an increase in the shear tensile strength for all types of adhesive bonds with alkali-treated filler compared to untreated filler. The shear tensile strength of adhesive bond C was 9.14 ± 0.71 MPa and that of adhesive bond C_NaOH was 12.84 ± 0.26 MPa, an increase of 41%. The adhesive bond J was 13.47 ± 0.39 MPa and that of the adhesive bond J_NaOH was 14.09 ± 0.98 MPa (an increase of 5%). The S adhesive bond was 11.96 ± 0.41 MPa, and the S_NaOH adhesive bond was 12.33 ± 0.41 MPa (increase of 3%). Compared to the AB0 adhesive bond, there was a significant increase in the shear tensile strength for all types of adhesive bond, that is, with and without modified filler. The value of the shear tensile strength for the AB0 adhesive bond was 7.96 ± 0.42 MPa, which is 15–69% less than the C, S, and J adhesive bonds and 55–77% less than the C_NaOH, J_NaOH, and S_NaOH adhesive bonds. The positive effect of the alkaline treatment of the natural filler NaOH on mechanical properties has also been confirmed by other authors [55,56].

The addition of a filler based on oil plant microparticles, which modifies the relatively fragile matrix, has also been shown to have a positive effect [57].

**Figure 7:**
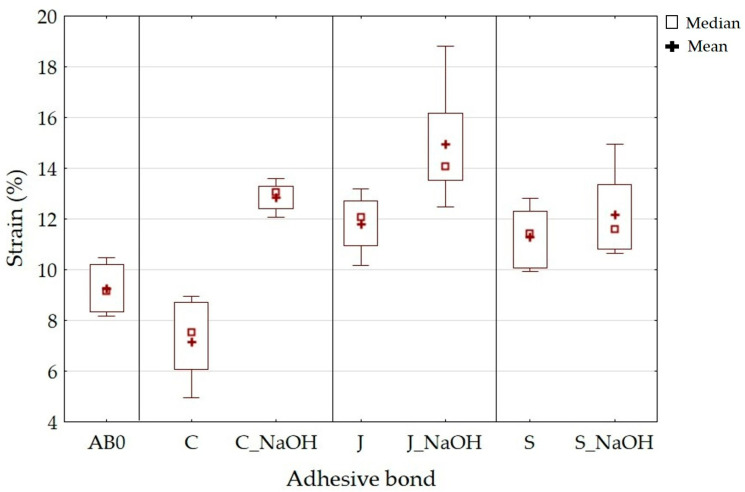
Static tensile test—strain.

The static strain results are shown in Figure 7. Adhesive bond C showed a strain of 7.15 ± 1.36%; adhesive bond J showed 11.79 ± 0.99% and adhesive bond S showed 11.30 ± 1.08%. Figure 6 shows that the adhesive bonds with alkaline filler treatment showed an increase in strain. The adhesive bond C_NaOH showed a strain of 12.86 ± 0.49%, J_NaOH 14.96 ± 1.96%, and S_NaOH 12.18 ± 1.41%. The above strain results along with the strength indicate good resistance to cyclic loading. The adhesive bond AB0 showed a strain of 5.14 ± 0.84%. Such low strain along with low strength indicates low resistance to cyclic loading, as shown by previous research [52].

The results of the statistical tests of static tensile strength and strain are shown in Table 4.

Table 4 shows that there is a statistically significant difference between adhesive bonds C and C_NaOH (*p* = 0.01). Thus, the alkaline treatment of the filler significantly affected the static tensile strength of the adhesive bond. Statistically insignificant differences were observed for all other types of adhesive bonds with the composite adhesive layer, that is, J and J_NaOH (*p* = 0.18), S and S_NaOH (*p* = 0.14). Thus, the alkaline treatment of the filler did not have a significant effect on the static tensile strength. The statistical evaluation of strain showed a significant effect between adhesive bonds C; C_NaOH (*p* = 0.01) and J; J_NaOH (*p* = 0.01). A statistically insignificant difference was shown for adhesive bonds S; S_NaOH (*p* = 0.25). In the comparison of the static tensile strength and strain of the AB0 adhesive bond and with the composite adhesive bonds, a statistically significant difference was evident (*p* = 0.01). This shows that both the untreated and treated filler in NaOH had a significant effect on the mechanical properties of the adhesive bonds.

**Figure 8:**
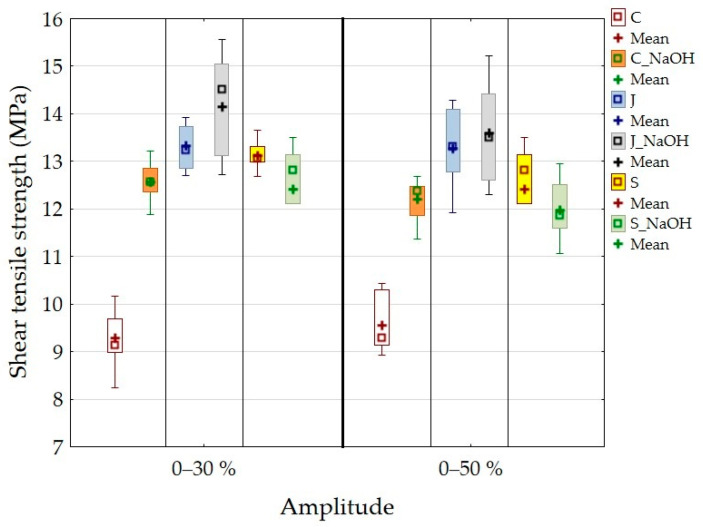
Strength results after cyclic loading of adhesive bonds with a composite adhesive layer.

The strength results after the cyclic loading of adhesive bonds with a composite adhesive layer are shown in Figure 8.

Figure 8 shows that the strength of the adhesive bond C is 9.26 ± 0.57 MPa at a cyclic loading amplitude of 5–30%. The adhesive bond C_NaOH showed a strength of 12.57 ± 0.38 MPa after cyclic loading. When comparing the static shear strength and the amplitude of 5–30%, there was a slight increase in strength of 1% for adhesive bond C and a decrease in strength of 2% for adhesive bond C_NaOH. Adhesive bond J showed a strength of 13.32 ± 0.42 MPa, and adhesive bond J_NaOH showed a strength of 14.15 ± 1.05 MPa. Compared to the static test, there was a 1% reduction in strength for adhesive bond J and a 0.4% increase in strength for adhesive bond J_NaOH. Adhesive bond S showed a strength of 13.13 ± 0.28 MPa, and adhesive bond S_NaOH showed a strength of 12.41 ± 1.15 MPa. Compared to the static test, there was an increase in strength of 10% for adhesive bond S and an increase of 4% for adhesive bond S_NaOH. The above results of the cyclic test with an amplitude of 5–30% indicate that the alkaline treatment of the filler surface does not have a significant effect on the shear strength of the adhesive bonds. Statistical testing confirmed this fact (*p* > 0.05).

The adhesive bond C showed a strength of 9.56 ± 0.55 MPa, and the adhesive bond C_NaOH showed a strength of 12.21 ± 0.41 MPa under cyclic loading with an amplitude of 5–50%. Compared to the static shear strength, the strength is 4.6% higher for adhesive bond C and 5% lower for adhesive bond C_NaOH. Adhesive bond J showed a strength of 13.27 ± 0.73 MPa and adhesive bond J_NaOH 13.59 ± 0.94 MPa after cyclic testing at 5–50% amplitude. Compared to the static test, there was a 1.5% and 3.5% reduction in strength for adhesive bond J and adhesive bond J_NaOH, respectively. Adhesive bond S showed a strength of 13.05 ± 0.44 MPa, and adhesive bond S_NaOH showed a strength of 11.97 ± 0.58 MPa. Compared to the static test, there was a 9% increase in strength for adhesive bond S, and the strength of adhesive bond S_NaOH was identical to the static test. From the above results of the cyclic test with an amplitude of 5–50%, the alkaline treatment of the filler surface does not have a significant effect on the shear strength of the adhesive bonds. Statistical testing confirmed this fact (*p* > 0.05).

When comparing the results of cyclic tests with different amplitudes, that is, between 5–30% and 5–50%, a difference in strength values is evident. At the higher cyclic loading amplitude, that is, 5–50%, there was a slight reduction in strength, namely 0.2% for adhesive bond J and a reduction of 4% for adhesive bond J_NaOH. There was a reduction of 0.6% for S adhesive bond and a reduction of 3.5% for S_NaOH adhesive bond. Thus, a negative effect of higher values of cyclic stress is evident, which causes a deterioration of the mechanical properties, that is, the strength of the adhesive bonds.

**Figure 9:**
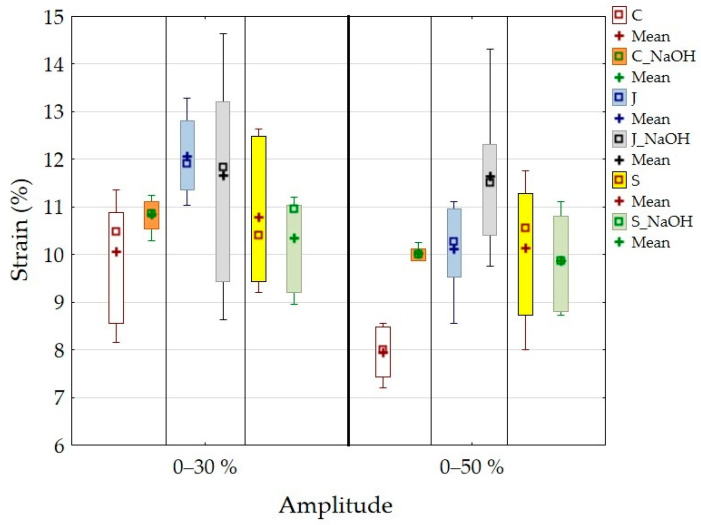
Strain results after cyclic loading of adhesive bonds with a composite adhesive layer.

The strain results after the cyclic loading of the adhesive bonds with the composite adhesive layer are shown in Figure 9.

Figure 9 shows a strain of 10.06 ± 1.12% for adhesive bond C and 10.84 ± 0.33% for adhesive bond C_NaOH at a cyclic loading amplitude of 5–30%. Compared to the static test, the strain increased by 40% for adhesive bond C and decreased to 16% for adhesive bond C_NaOH. Adhesive bond J showed a strain of 13.32 ± 0.42%, and adhesive bond J_NaOH showed a strain of 14.15 ± 1.05%. Compared to the static test, the strain increased by 11% for adhesive bond J and decreased by 6% for adhesive bond J_NaOH. The adhesive bond S showed a strain of 10.79 ± 1.25%, and the adhesive bond S_NaOH showed a strain of 10.34 ± 0.88%. Compared to the static test, the strain decreased by 5% for the adhesive bond S and decreased by 15% for the adhesive bond S_NaOH. Statistical testing showed a statistically non-significant difference for adhesive bond J_NaOH and adhesive bond S (*p* > 0.05), that is, no effect of alkali treatment on strain at a cyclic stress amplitude of 5–30% was demonstrated. For all other types of adhesive bonds, a statistically significant difference (*p* < 0.05) was demonstrated, that is, an effect of alkali treatment on strain was demonstrated at a cyclic loading amplitude of 5–30%.

The results of cyclic tests of the AB0 adhesive bond are not comparable with the results of the adhesive bonds with a composite adhesive layer due to the different values of the maximum force from the static test (2470 N), that is, also the values at amplitudes of 5–30, 50% (123–741, 1235 N). Research is not aimed at comparing the adhesive bond AB0 with the adhesive bonds with the composite adhesive layer under cyclic loading. However, the AB0 adhesive bond showed a 13% higher strength of 9.03 ± 1.04 MPa and the strain of 8.94% under cyclic loading of 5–30% amplitude. ∆Strain was 0.09%. Cyclic loading of 5–50% amplitude caused the premature failure of the AB0 adhesive bond, that is, AB0 did not withstand the required 1000 cycles. The number of completed cycles was 238 ± 15. From these results of the adhesive bond, it can be concluded that premature failure occurred at higher cyclic loading amplitudes, even with a relatively small number of completed cycles [58]. Therefore, it can be concluded that the filler in the adhesive layer positively influenced the service life of the adhesive bonds.

Table 5 presents the evaluation of cyclic loading tests in terms of ∆Strain, finished tests, and number of finished cycles. These parameters are significant in terms of the service life of adhesive bonds under cyclic loading. Table 5 shows a considerable influence of the amplitude of cyclic loading on ∆Strain. The average ∆Strain was 0.09% at an amplitude of 5–30% and 0.70% at an amplitude of 5–50%. Increasing ∆Strain indicates the progressive fatigue of adhesive bonds. The fatigue process is related to the cumulative damage of cyclic loading, which results, for example, in the premature failure of adhesive bonds [59]. It can be concluded from the above that the higher the amplitude of cyclic loading, the earlier the premature failure of the adhesive bonds. The above phenomenon has been demonstrated by studies dealing with higher cyclic loading amplitudes up to 70% of the maximum force [36,46,52]. However, there are noticeable differences in ∆Strain between adhesive bonds with treated and untreated filler, especially at higher cyclic loading amplitudes of 5–50%. The adhesive bonds C, J, and S showed an average ∆Strain of 0.76%, and the adhesive bonds C_NaOH, J_NaOH and S_NaOH showed 0.64%. Therefore, it is evident that the alkaline filler treatment led to a reduction in ∆Strain and therefore to the increase in the service life of the adhesive bonds under cyclic loading. At an amplitude of 5–30%, no significant difference in ∆Strain between treated and untreated filler was demonstrated; see Table 5. All types of adhesive bonds with the composite adhesive layer withstood the specified 1000 cycles. Broughton et al. [60] report that an amplitude of 50% is used in the aerospace industry to determine safety factors in the design of adhesive-bonded and bolted structures under cyclic loading.

**Figure 10:**
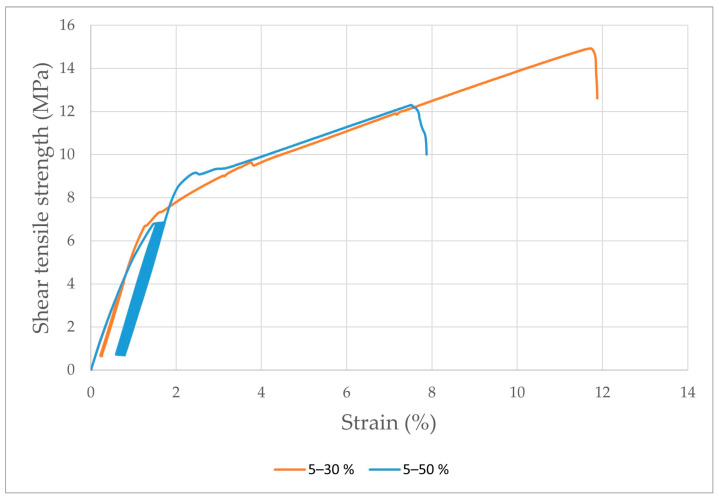
Quasi-static curves of the J_NaOH adhesive bond at a cyclic loading amplitude of 5–30% and 5–50%.

Examples of quasi-static curves are shown in Figure 10. Figure 10 shows the difference in ∆Strain between the 1st and 1000th cycles at different cyclic loading amplitudes.

Table 6 shows the evaluation of the types of failure of each type of adhesive bond. All types of adhesive bonds showed a predominantly adhesive–cohesive type of failure after static testing. The adhesive type of failure was minimally represented (for adhesive bonds C and C_NaOH), which may have been due to the inadequate preparation of the bonded surface. Table 6 shows the difference between the failure types for adhesive bonds without filler surface treatment and with surface treatment in 5% NaOH solution under the cyclic loading of different amplitudes. Adhesive bonds C, J, and S showed a predominantly adhesive type of failure under cyclic loading. In this case, the adhesive failure could have been caused by the modification of the bonded layer study relating to the use of coconut shells as a particulate filler [47]. The adhesive bonds C_NaOH, J_NaOH, and S_NaOH showed the predominantly adhesive–cohesive type of failure under cyclic loading. Thus, it is evident that the filler surface treatment affected the failure type of these adhesive bonds under cyclic loading.

SEM analysis is therefore an important and universal tool for checking the structure of the materials to be tested and, in the case of composite-based materials, the interface between the filler and the matrix [61].

**Figure 11:**
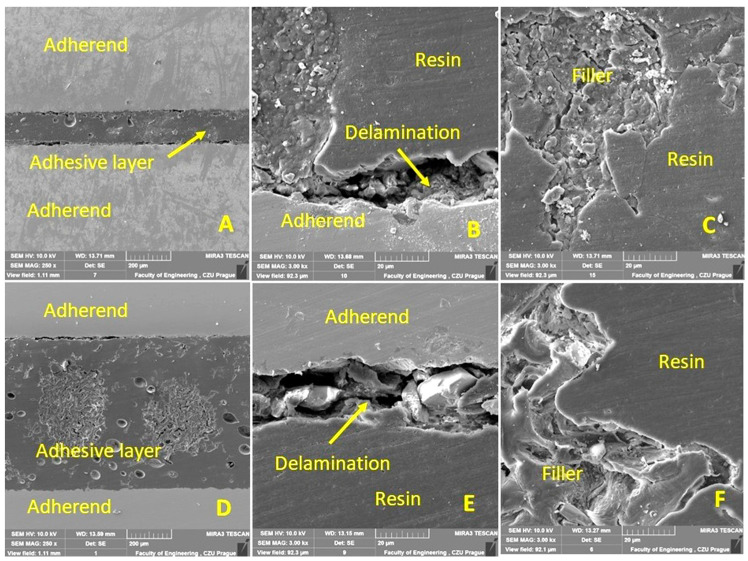
SEM analysis of a cross-section of a hybrid adhesive layer with C-filler (SE detector Oxford): (**A**): Cross-section of a hybrid adhesive bond with C-filler (MAG 250×), (**B**): Interaction between hybrid adhesive layer and adherend (MAG 3000×), (**C**): Interaction between C-filler and resin (MAG 3000×), (**D**): Cross-section of hybrid adhesive bond with filler C_NaOH (MAG 250×), (**E**): Interaction between hybrid adhesive layer and adherend (MAG 3000×), (**F**): Interaction between filler C_NaOH and resin (MAG 3000×).

**Figure 12:**
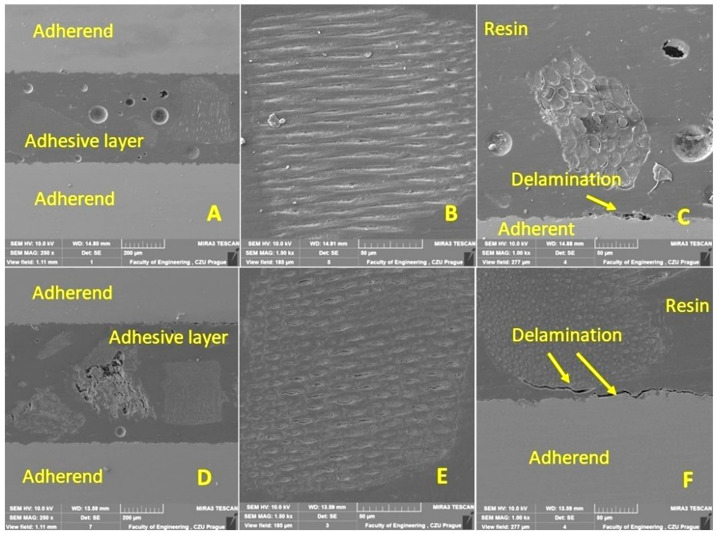
SEM analysis of a cross-section of a hybrid adhesive layer with S-filler (SE detector Oxford): (**A**): Cross-section of a hybrid adhesive layer with S-filler (MAG 250×), (**B**): Interaction between S-filler and resin (MAG 1500×), (**C**): Interaction between hybrid adhesive layer and adherend (MAG 1000×), (**D**): Cross section of a hybrid adhesive bond with S_NaOH (MAG 250×), (**E**): Interaction between S_NaOH (MAG 1500×), (**F**): Interaction between hybrid adhesive layer and adherend (MAG 1000×).

**Figure 13:**
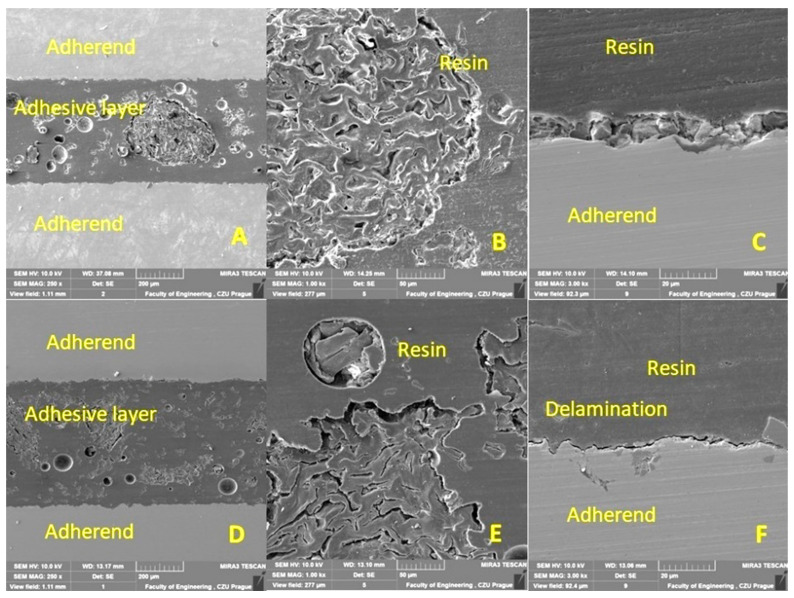
SEM analysis of the cross-section of the hybrid adhesive layer with J filler (SE detector Oxford): (**A**): Cross section of the hybrid adhesive layer with filler J (MAG 250×), (**B**): Interaction between J filler and resin (MAG 1000×), (**C**): Interaction between hybrid adhesive layer and adherend (MAG 3000×), (**D**): Cross-section of hybrid adhesive bond with filler J_NaOH (MAG 250×), (**E**): Interaction between filler J_NaOH and resin (MAG 1000×), (**F**): Interaction between hybrid adhesive layer and adherend (MAG 3000×).

**Figure 14:**
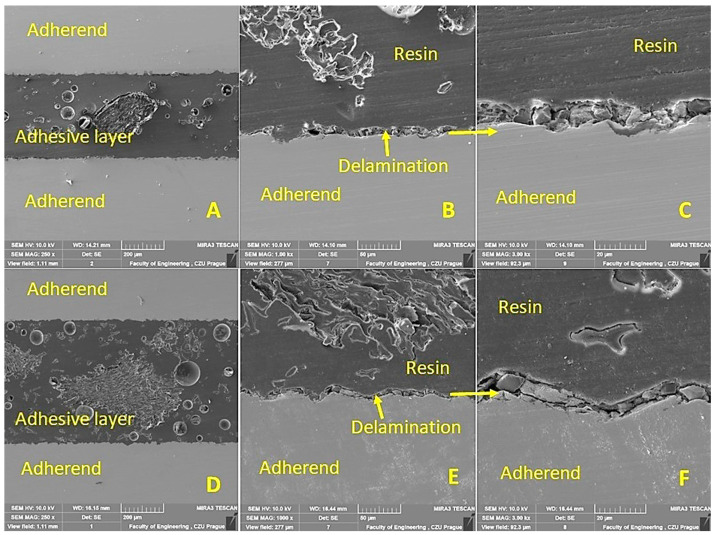
SEM analysis of a cross section of a hybrid J-filler adhesive layer after 1000 cycles at a load cycle test of 5–50% (Oxford SE detector): (**A**): Cross-section of hybrid adhesive bond with J-based filler (MAG 250×), (**B**): Interaction between adherend and hybrid adhesive layer (MAG 1000×), (**C**): Detail of the interaction between the hybrid adhesive layer and the adherend (MAG 3000×), (**D**): Cross-section of hybrid adhesive bond with filler J_NaOH (MAG 250×), (**E**): Interaction between adherend and hybrid adhesive layer (filler J_NaOH and resin) (MAG 1000×), (**F**): Detail of the interaction between the hybrid adhesive layer and adherend (MAG 3000×).

The cohesion mechanism of the adhesive bond is dependent on the adhesion, the wettability of the adhesive, and its own cohesion, which significantly affect the resulting strength of the adhesive bond. In terms of internal structure, any structurally strong and sufficiently durable adhesive bond of two basic materials can be considered a complex of three main layers, namely the adhesive material, the adhesive layer, and the cohesive layer. The characterization of the hybrid adhesive bonds and the individual layers while considering the interaction of the filler with the matrix was performed by SEM. The results of the SEM analysis are presented in Figure 11, Figure 12, Figure 13 and Figure 14. Figures show a cross-section of the hybrid adhesive layer and the interaction of the individual layers.

Over the past few decades, natural fillers have become a sought-after commodity in the field of composite materials. The reason for the increased interest is mainly due to the demand for environmentally friendly materials or products that contain a proportion of environmentally friendly materials [62].

A problematic aspect of these natural fillers is their low interaction with the matrix, or delamination within the hybrid adhesive layer. These limits can usually only be determined by the SEM analysis of the fracture surface or cross section through the tested layer [63,64,65].

The possible improvement in the adhesion of the oilseed filler can be improved by chemical treatment, as highlighted by Hasanah et al. [66] and Gae et al. [67] in their research on coconut shells. The positive effect on the mechanical properties and service life of adhesive bonds under cyclic loading was confirmed by the research of various microparticles in the form of fillers added to the adhesive bonds due to the better stress distribution in the matrix [47]. Even the filler based on crushed coconut-shell microparticles was characterized by considerable shape variability, reduced degree of filler–matrix wetting, and the low interaction of irregular filler and matrix [47]. Comparable results can be seen in Figure 11B,C,F, Figure 12D,F, and Figure 13B,E.

From the cross sections (Figure 11, Figure 12, Figure 13 and Figure 14), the bubbled structure of the adhesive layer was characterized by porosity. The adhesive bonds using filler from Jatropha Curcas L. are characterized by a bubbled structure inside the adhesive layer. Porosity is of varied sizes, that is, 11 ± 4 µm. These bubbles (porosity) are characterized by varied sizes ranging from 7 to 15 µm. Most bubbles (porosity) are smaller than 11 µm. The results also show the different thicknesses of the adhesive layer, which were 149 ± 5 µm for the adhesive bond C, 685 ± 5 µm for the adhesive bond C_NaOH, 439 ± 5 µm for the adhesive bond J, 527 ± 6 µm for the adhesive bond J_NaOH, 422 ± 4 µm for the adhesive bond S, and 573 ± 6 µm for the adhesive bond S_NaOH.

Figure 11 shows the results of the SEM analysis of a cross-section of adhesive bonds with filler C. Figure 11A–C uses filler C (without chemical treatment). Figure 11A shows significant porosity within the adhesive layer. Figure 11B shows delamination at the interface of the adherend and the hybrid adhesive layer. This is a weak adhesion. Figure 11C shows the interaction of filler C with the resin. Figure 11D–F presents the results of the interaction between each layer of the adhesive bond and the filler C_NaOH (treated in the NaOH solution). Significant porosity (Figure 11D) and delamination between the adherend and the resin (Figure 11E) are also evident. The specific irregular shape and texture of filler C are evident from Figure 11C,F. Filler C_NaOH showed better wettability with resin.

Figure 12 shows the results of the SEM analysis of the cross section of adhesive bonds with filler S. Figure 12A–C uses filler S (without chemical treatment). Figure 12A also shows significant porosity within the adhesive layer. In Figure 12B, a good interaction of the filler S with the resin is evident. From Figure 12C, delamination at the interface of the adherend and hybrid adhesive layer is evident, which significantly reduces adhesion. Figure 12D–F presents the results of the interaction between each layer of the adherend and the filler S_NaOH (treated in the NaOH solution). Significant porosity (Figure 12D) and delamination between the adherend and resin (Figure 12F) were also evident. A specific irregular shape and texture of filler S are evident from Figure 12B,C,E,F. A dual type of filler are the skin (Figure 12B,E) and the kernel fillers presented in Figure 11 and Figure 13. These were residual particles associated with the base of the skin. For filler S_NaOH, no significant improvement in wettability with resin was demonstrated (Figure 12F).

Figure 13 shows the results of the SEM analysis of the cross section of the adhesive bonds with filler J. Figure 13A–C uses filler J (without chemical treatment). Again, Figure 13A shows significant porosity within the hybrid adhesive layer. Figure 13B shows the poor interaction of filler J with the resin, which significantly reduces the cohesive strength of the hybrid adhesive layer. Figure 13C shows significant delamination at the interface between the adherend and the hybrid adhesive layer, which significantly reduces the strength of the adhesive bond. Figure 13D–F presents the results of the interaction between each layer of the adherend and the filler J_NaOH (treated in NaOH solution). Significant porosity (Figure 13D) and delamination at the interface of the filler and resin (Figure 13E) and the adherend and hybrid adhesive layer (Figure 13F) were also observed. Figure 13B,E shows the specific irregular shape and texture of filler J, characterized by porosity. Even for the filler J_NaOH, no significant improvement in the wettability with resin was demonstrated (Figure 13F).

Figure 14 again presents the results of the SEM analysis of hybrid adhesive bonds with filler J. A cross section of the adhesive bond was prepared from test bodies subjected to 1000 cycles in a load cycle test of 5–50%. It can be noted that no difference was found between the results of SEM analysis in the section of the test bodies before and after the load cycle test of 5–50%. Figure 14A–C uses filler J (without chemical treatment). Figure 14A again shows significant porosity within the hybrid adhesive layer. Figure 14B shows the poor interaction of filler J with the resin, which again can result in a significant reduction in the cohesive strength of the hybrid adhesive layer. Figure 14C shows significant delamination at the interface between the adherend and the hybrid adhesive layer at various magnifications, significantly reducing the adhesion. Figure 14D–F presents the results of the interaction between each layer of the adherend and the J_NaOH filler. Significant porosity (Figure 14D) and delamination at the interface of the filler and resin (Figure 14E,F) and the adherend and hybrid adhesive layer (Figure 14F) were also observed.

## 4. Conclusions

There are studies that deal with the alkali treatment of the surface of natural fillers and its effect on the mechanical properties of newly formed composites. However, these studies are largely focused on the evaluation of static mechanical characteristics, such as static tensile strength, etc. This research is focused on the evaluation of the effect of the alkaline treatment of the surface of natural oil-based fillers on the cyclic loading service life of adhesive bonds. The adhesive bonds have the characteristic of having low resistance to cyclic loading. Cyclic loading is a common cause of the premature failure of structural bonds, including adhesive bonds, and it is therefore the subject of further research to find solutions that minimize its negative impact. The use of natural fillers, which is the current research trend in polymer composites, can be a solution to increase the service life of adhesive bonds under cyclic loading, and thus ways to use them effectively must be sought. The material utilization of all the products generated and, therefore, the waste can help not only from an environmental perspective but also from an economic perspective.

The results of experiments on hybrid adhesive bonds with untreated and in 5% aqueous NaOH solution-treated surfaces of filler obtained after the pressing of Jatropha Curcas L. (oilseed cakes) showed:

- Increase in the static shear strength of the C_NaOH, J_NaOH, and S_NaOH adhesive bonds in the range of 3–41% compared to the C, J, and S adhesive bonds;
- A positive effect of the addition of a filler based on oil plant microparticles, which modifies the fragile matrix;
- Statistically insignificant differences in the effect of alkali treatment on the shear strength of adhesive bonds with a composite adhesive layer at cyclic stress amplitudes of 5–30% and 5–50% (*p* > 0.05). A statistically insignificant difference was found in the effect of alkali treatment on strain, for S and J_NaOH adhesive bonds at a cyclic stress amplitude of 5–30%. Statistically significant differences (*p* < 0.05) were observed for all other types, including adhesive bonds loaded at an amplitude of 5–50%;
- A negative effect of cyclic loading at a higher amplitude of 5–50% on shear strength compared to 5–30%. At a cyclic loading amplitude of 5–50%, there was a slight reduction in strength, 0.2% for J adhesive bonds and 4% for J_NaOH adhesive bonds. There was a reduction of 0.6% for S adhesive bonds and a reduction of 3.5% for S_NaOH adhesive bonds;
- Noticeable differences in ∆Strain between adhesive bonds with modified and unmodified filler, especially at higher cyclic stress amplitudes of 5–50%. The adhesive bonds C, J, and S showed an average ∆Strain of 0.76%, and the adhesive bonds C_NaOH, J_NaOH and S_NaOH showed 0.64%. Therefore, it is evident that alkaline filler treatment led to a reduction in ∆Strain and therefore an increase in the lifetime of the adhesive bonds under cyclic loading. At an amplitude of 5–30%, no significant difference in ∆Strain between treated and untreated filler was demonstrated;
- Based on SEM analysis fillers based on the kernel without skin from the Jatropha Curcas L. designated (J, J_NaOH) and the whole kernel including skin (C, C_NaOH) have low interaction with the matrix (resin) due to the higher oil content. SEM analysis showed no difference in the interaction between the hybrid adhesive layer and the bonded material (adherend) for any variant of the experiment, differing by filler type. Delamination also occurred before the load cycle test. The SEM analysis showed the presence of interlayer defects in the fracture of the tested materials, which are related to the oilseed plant filler used.

## Figures and Tables

**Table 1:** Marking and characteristics of fillers (adhesive bonds).

Marking	Characteristics
C	Whole kernel including skin; without alkali treatment
S	Skin; without alkaline treatment
J	Kernels without skin; without alkaline treatment
C_NaOH	Whole kernel including skin; alkaline treatment in 5% NaOH
S_NaOH	Skin; alkaline treatment in 5% NaOH
J_NaOH	Kernels without skin; alkaline treatment in 5% NaOH
AB0	Pure adhesive; no filler

**Table 2:** Basic mechanical properties of S235J0 steel at 20 °C [53].

Tensile Strength	340–470 MPa
Yield Strength	225–235 MPa
Elastic Modulus	212 GPa
Elongation	24%

**Table 3:** Setup of cyclic tests based on static test results.

Adhesive Bond	Fmax of C, S, J (N)	Amplitude 5–30% of Fmax of C, S, J (N)	Amplitude 5–50% of Fmax C, S, J (N)
C; C_NaOH	2810	140–843	140–1405
J; J_NaOH	4246	212–1274	212–2123
S, S_NaOH	3726	186–1118	186–1863

**Table 4:** Statistical evaluation of static tensile test at 0.05 level of significance-ANOVA F-test.

*p*-Parameter						
	Tensile Strength			Strain		
	C; C_NaOH	J; J_NaOH	S; S_NaOH	C; C_NaOH	J; J_NaOH	S; S_NaOH
	0.01	0.18	0.14	0.01	0.01	0.25
AB0	0.01; 0.01	0.01; 0.01	0.01; 0.01	0.01; 0.01	0.01; 0.01	0.01; 0.01

**Table 5:** Evaluation of cyclic loading tests of adhesive bonds with a composite adhesive layer.

Amplitude	Adhesive Bond Type	∆Strain (1st–1000th Cycle)	Finished Tests
		[%]	[-]
5–30%	C	0.16	7/7
	J	0.09	7/7
	S	0.08	7/7
	C_NaOH	0.08	7/7
	J_NaOH	0.08	7/7
	S_NaOH	0.08	7/7
5–50%	C	0.79	7/7
	J	0.75	7/7
	S	0.76	7/7
	C_NaOH	0.67	7/7
	J_NaOH	0.63	7/7
	S_NaOH	0.62	7/7

**Table 6:** Evaluation of the type of failure of individual types of adhesive bonds.

Type of Adhesive Bond	Characteristics of the Adhesive Bond Test	AF ^1^	A/CF ^2^
C	Static test	2	5
	Amplitude 5–30%	6	1
	Amplitude 5–50%	4	3
J	Static test	0	7
	Amplitude 5–30%	5	2
	Amplitude 5–50%	2	5
S	Static test	0	7
	Amplitude 5–30%	7	0
	Amplitude 5–50%	4	3
C_NaOH	Static test	1	6
	Amplitude 5–30%	2	5
	Amplitude 5–50%	1	6
J_NaOH	Static test	0	7
	Amplitude 5–30%	0	7
	Amplitude 5–50%	2	5
S_NaOH	Static test	0	7
	Amplitude 5–30%	3	4
	Amplitude 5–50%	1	6

^1^ Adhesive failure; ^2^ Adhesive/cohesive failure.

## Data Availability

Not applicable.
